# Epidemiology of Pertussis After the COVID-19 Pandemic: Analysis of the Factors Involved in the Resurgence of the Disease in High-, Middle-, and Low-Income Countries

**DOI:** 10.3390/vaccines12121346

**Published:** 2024-11-28

**Authors:** Lucia F. Bricks, Juan C. Vargas-Zambrano, Denis Macina

**Affiliations:** 1 Sanofi Vaccines Medical, Av Nações Unidas, São Paulo 14401, Brazil; 2 Sanofi Vaccines Medical, 14 Espace Henri Vallee, 69007 Lyon, France; juan.vargas@sanofi.com (J.C.V.-Z.); denis.macina@sanofi.com (D.M.)

**Keywords:** pertussis, vaccines, epidemiology, diagnosis, surveillance

## Abstract

Pertussis is a highly contagious bacterial disease of the respiratory tract that can be prevented by vaccination. Before the COVID-19 pandemic, the vaccine coverage rate for the third dose of a DPT-containing vaccine was 86%, with large disparities among countries. Since 2022, many high-income countries have reported a resurgence of pertussis, especially in the European region, but the disease has also caused outbreaks in middle- and low-income countries, despite their less extensive disease surveillance capacities. This paper aims to review the incidence rates (IRs) of pertussis in high-, middle-, and low-income countries following the COVID-19 pandemic and to discuss the most relevant factors associated with the resurgence of the disease. The epidemiology of pertussis is highly variable and is influenced not only by the type of vaccine used but also by the specific characteristics of the disease, vaccine coverage rates, vaccination schedules, and the quality of surveillance. Since the cessation of COVID-19 measures, there have been significant pertussis outbreaks that could have been partially mitigated with higher coverage rates and more comprehensive pertussis vaccination throughout life.

## 1. Introduction

Pertussis, commonly known as whooping cough, is a highly contagious respiratory disease caused by *Bordetella pertussis*, a Gram-negative bacillus that exclusively infects humans. The bacterium has a limited survival time outside its host, contributing to its human-specific transmission. Pertussis occurs globally in both endemic and epidemic forms, affecting individuals of all ages. However, its incidence is highest among unvaccinated or incompletely vaccinated infants and young children, who are particularly vulnerable to severe disease and complications. Infants under six months old, particularly those who are premature or have low birth weight, face the greatest risk, with an elevated likelihood of complications, hospitalization, and even death [1,2,3,4].

Transmission occurs through respiratory secretions and the disease is known for its high infectivity, with a basic reproduction number (R_0_) ranging from 12 to 17, comparable to that of measles. This high infectivity, combined with a low infectious dose, makes pertussis highly transmissible, particularly in crowded environments such as schools and universities [1,2,3].

The peak period of transmissibility occurs within the first three weeks after the onset of coughing, during which the risk of transmission is highest. Although breakthrough infection in previously vaccinated individuals generally proceeds to milder symptoms, complications can still arise in up to 25% of cases [1,2,3]. Importantly, recent studies suggest that asymptomatic carriers may also contribute to the spread of the disease, further complicating diagnosis and control efforts [5,6].

Despite the availability of vaccines, pertussis is not considered eradicable due to its high transmissibility and the waning of immunity that follows both natural infection and vaccination. Vaccines provide incomplete and non-sterilizing immunity, making booster doses necessary throughout life to maintain protection [1,2,7]. The primary aim of vaccination is to reduce the disease burden in young infants, with the World Health Organization (WHO) recommending three primary doses in infancy followed by periodic boosters. Since 2015, maternal vaccination during pregnancy has been advised to protect newborns under three months of age [2].

Globally, vaccine coverage rates (VCRs) have improved over the past two decades, reaching 86% for the third dose of diphtheria-, tetanus-, and pertussis-containing vaccines (DPT) in 2018. However, the COVID-19 pandemic disrupted immunization programs, causing a decline to 83% by 2021. In 2023, the VCR for the third dose of DTP was 84%, but 14.5 million infants did not receive an initial dose of a DTP vaccine and an additional 6.5 million are partially vaccinated. Of these 21 million, just under 60% of these children live in 10 countries: Afghanistan, Angola, the Democratic Republic of the Congo, Ethiopia, India, Indonesia, Nigeria, Pakistan, Sudan, and Yemen [3]. In several of these countries, VCRs remain below 90% [8] and in some, booster doses after the first year of life are not routinely administered [9]. Interestingly, despite lower VCRs in many low- and middle-income countries, most recent pertussis outbreaks have been reported in developed nations [8,10].

This paper aims to review the incidence of pertussis following the COVID-19 pandemic and explore key factors contributing to its resurgence, based on the WHO and Ministry of Health websites of countries that reported pertussis outbreaks and the most recent literature available in PUBMED.

## 2. Results

### 2.1. Global Epidemiology of Pertussis Pre- and Post-COVID-19 Pandemic

Between 2019 and 2022, during the COVID-19 pandemic, there was a marked reduction in the incidence rate (IR) of pertussis. This decrease has been attributed to widespread public health measures, such as the use of masks, social isolation, enhanced hygiene practices, and disruptions in healthcare access and disease reporting [3]. According to the WHO, the global pertussis IR fell four-fold from 16.1 per million in 2016 (pre-pandemic) to 4.6 per million in 2021. However, starting in 2023, the global IR began to rise again, reaching 10.2 per million. Notably, this resurgence was not uniform across all regions. In Europe, the IR increased 30-fold between 2021 and 2023, although significant variation was observed across regions (Table 1) and countries (Table 2) [10].

### 2.2. Pertussis Incidence Rates in High-Income Countries

High-income countries (HICs) consistently report higher incidence rates than middle-income countries (MICs) and low-income countries (LICs) [10].

Table 2 shows the incidence rates of pertussis in HICs that experienced an increase in cases during the post-pandemic period. All of these countries use acellular pertussis (aP) vaccines, albeit with different vaccination schedules, and report high vaccine coverage rates (VCRs) [8,9,10]. The pertussis IRs in Austria, Croatia, and Denmark were the highest in the last 10 years. In Belgium and Croatia, the pertussis VCRs were not impacted by the COVID-19 pandemic, and in Austria, since 2018, before the COVID-19 pandemic, the VCRs varied between 84–86% [8].

According to the European Centre for Disease Prevention and Control (ECDC), pertussis cases surged dramatically in Europe, with over 32,000 cases and 19 deaths reported between January and April 2024. However, the rise in cases was uneven across the continent, initially affecting Austria, Denmark, and Norway in the second half of 2023 [11]. Variations in incidence across age groups were influenced by factors such as vaccination schedules (2 + 1 versus 3 + 1 in the primary series), the timing and frequency of booster doses, availability of laboratory diagnostics, and the surveillance strategies in place [7,11,12,13,14,15,16,17,18,19].

In Europe, the USA, and Australia, the majority of cases were recorded among adolescents, adults, and elderly populations [7,11,12,13,14,15,16,17,18,19,20,21,22]. In Canada [23,24], Spain [15], and Czechia [17], outbreaks primarily affected children over five years of age and adolescents, particularly in areas where VCRs were below 90%.

Additionally, in some communities, particularly those with vaccine hesitancy, outbreaks occurred despite high national VCRs. For instance, in Israel, where national VCR exceeds 90%, an outbreak occurred in a religious community that refuses vaccination, primarily affecting infants under one year of age [25].

Partial data from 2024 indicate that pertussis continues to spread in developed countries. Notable outbreaks were recorded in the UK (10,493 cases) [14], Czechia (7898) [17], the USA (22,273) [20], France (>34,000 cases) [17,18], and Australia (30,544 cases) [21].

Despite most cases occurring among older individuals, the highest mortality rates were seen in young infants. In Europe, 19 deaths were recorded between January and March 2024, with 58% in infants and 42% in people aged ≥ 60 years [11]. The case-fatality rate was very low (0.056%). In HICs, where the VCR for the third dose of DTP-containing vaccines is high, there is an increasing emphasis on vaccinating pregnant women with Tdap to protect infants under three months of age [11,12,13,14].

### 2.3. Pertussis Incidence Rates in Middle- and Low-Income Countries

In many MICs and LICs, a resurgence of pertussis was also noted after the COVID-19 pandemic. Most LICs use whole-cell pertussis (wP) vaccines with few or no booster doses, and there are limited data available on pertussis incidence in these regions. Some MICs, while still primarily using wP vaccines, have introduced aP vaccines in their schedules. Table 3 presents data on MICs and LICs that reported higher pertussis IRs during the post-pandemic period. Afghanistan, Bolivia, China, Ecuador, Indonesia, Malaysia, Papua New Guinea, the Russian Federation, and Serbia reported their highest pertussis IRs since 2014 [9,10].

The situation of LICs and MICs is diverse and detailed data are not available for most countries; however, some pertussis outbreaks were reported in several of these countries, independently of the type of pertussis vaccine used in routine [9,10].

Among these countries using aP vaccines, South Africa was one of the first countries to report a resurgence of pertussis after the COVID-19 pandemic. A total of 818 pertussis cases were reported between June 2022 and January 2023, mostly in Cape Town [26], but the highest IRs were observed in Russia and Serbia [10,27,28]. Serbia reported more than 1400 pertussis cases, the highest number the last two decades [28]. China, where aP co-purified vaccines have been used since 2015, recorded a dramatic increase in pertussis cases over the past two years [29,30]. In April 2024, China recorded 91,272 cases of whooping cough and seven deaths [30].

Pertussis outbreaks were also registered in countries using wP vaccines. Despite the limited information about pertussis in the poorest countries, it is evident that even in some of these countries, pertussis is resurging. Two examples are Afghanistan and Papua New Guinea. In Afghanistan, in 2023, 1531 pertussis cases were registered, more than 10 times greater in comparison with 2021 (108 cases) [10]. Other countries reported regional pertussis outbreaks. Bolivia recorded 693 pertussis cases in 2023, mostly in Santa Cruz Province [31,32]. In Argentina, 605 cases were reported in 2023, most of which were concentrated in the province of Salta [33]. In Peru, despite the low IR, the number of pertussis deaths in 2023 was 144 with 6 deaths, a case-fatality rate (4.2%) higher than that observed in HICs [34]. In Brazil, despite the high number of children that did not receive any pertussis-containing vaccine in recent years, until 2023, the pertussis IR was only 1 per 1,000,000 in the population. However, since January 2024, pertussis outbreaks have been registered in many cities, with a total of 2954 confirmed pertussis cases and 12 deaths as of November 2024 [35].

## 3. Discussion

The high incidence rates of pertussis recorded in Europe (Table 1) might surprise those unfamiliar with the diagnostic criteria and vaccination strategies used across different regions. Many European countries use acellular pertussis vaccines, maintain high VCRs, and recommend booster doses for various groups, including schoolchildren, adolescents, adults, pregnant women, and healthcare professionals [8,9]. In contrast, lower IRs are observed in countries that primarily use whole-cell pertussis vaccines and often report lower VCRs and fewer boosters [8,9]. Several factors contribute to the resurgence of pertussis; notably, the specific characteristics of the disease, the type of vaccine used, VCRs, vaccination schedules, and the quality of epidemiological surveillance.

### 3.1. Specificities of the Disease

Pertussis is a highly transmissible disease that persists in both endemic and epidemic forms, with outbreaks occurring every 3–5 years, regardless of vaccination type or VCR [1,2,3]. The introduction of wP vaccines in the 1940s significantly reduced the peaks and frequency of epidemics, but these outbreaks never fully disappeared [1,2]. Similarly, countries that adopted aP vaccines following decreased acceptance of wP vaccination in the late 1970s saw continued outbreaks [7]. Pertussis is not eradicable because neither natural infection nor vaccination provides lifelong protection. Immunity wanes over time, and unless regular boosters perpetuate protection, the growing pool of susceptible individuals increases the likelihood of outbreaks [1,2,3,7,10,20,23].

### 3.2. Vaccine Coverage Rates and Vaccination Schedules

Globally, the highest IRs of pertussis are observed in unvaccinated or incompletely vaccinated infants. However, the age distribution of pertussis cases varies depending on VCRs and vaccination schedules. In HICs with high VCRs, most cases occur among adolescents and adults, including the elderly, but serious cases and deaths occurs more frequently in infants too young to receive the first doses of pertussis-containing vaccines [11,13,14,15,16,17,18,19,20,21,22,23,24]. In countries or areas with lower VCRs, children under five years of age are predominantly affected [1,2,8,9,36,37,38,39].

While HICs have maintained high VCRs for over two decades, they still experience periodic pertussis outbreaks [1,2,7]. Variable pertussis incidence rates were observed in MICs that adopted aP vaccines in their routine schedules, and this can be associated with VCRs. In Mexico, which reported a 72% VCR for the third dose of a DPT vaccine after the COVID-19 pandemic, there was a smaller increase in pertussis cases compared to Chile, which maintained VCRs above 90% even during the pandemic [10]. In China, where no booster vaccination is available beyond toddler age, most pertussis cases were observed in school-aged children [29,30]. Similarly, Russia, despite VCRs above 90% for the primary series, does not recommend booster doses for preschool children and has seen a surge in cases among school-aged children [27].

In MICs and LICs that primarily use wP vaccines, lower VCRs and low pertussis IRs might suggest that these vaccines offer better protection, but this is not the case. Underreporting is a big challenge in these countries where pertussis is still primarily viewed as a childhood disease and diagnostic capabilities are limited [10,36,37,38,39]. For example, countries like Angola, Bangladesh, Haiti, and Venezuela, which report low VCRs for diphtheria–tetanus–pertussis (DTP) vaccines (Table S1 in Supplementary Material), rarely report pertussis, despite experiencing other vaccine-preventable disease outbreaks, such as diphtheria or polio [8,10]. India and Indonesia have reported increases in pertussis IRs, but these figures are much lower than expected, given the number of unvaccinated children in these countries [10]. Argentina and Bolivia reported regional outbreaks despite the low VCRs in the entire country, showing that other factors, such as awareness and surveillance, are relevant factors related to epidemiologic information. In Brazil, in 2014 there was a pertussis outbreak with 8338 cases (IR = 41.7); from 2015 to 2019, the IRs varied from 6.9 to 14.5, despite the substantial reduction in VCR observed since 2015 (Table S1 Supplementary Material). From 2020 to 2023, the pertussis IRs ranged from 0.7 (2021) to 1.2 in 2022, but in 2024 the number of confirmed pertussis cases increased by almost 30 times (2954) as compared to 2023 (124), with large variation among the states. Parana State reported the highest number of cases (1108) and deaths (4 from 12), followed by São Paulo State (766). In these states, about 70% of cases were confirmed by laboratorial tests (culture and/or PCR) and more than 20% by clinical–epidemiological linkage. In the north and north-east region, low numbers of pertussis cases were reported (73), the majority were registered in children and those less than one year, and only 50% of pertussis cases have been confirmed by laboratory tests [35].

These examples demonstrate that pertussis resurgence is not solely dependent on the type of vaccine used (wP or aP). The WHO has emphasized that both vaccines are effective in preventing pertussis in the first year of life, but neither provides lifelong protection. Booster doses are essential to maintaining immunity and vaccination coverage should exceed 90% to reduce transmission risks [2].

### 3.3. Characteristics of Epidemiological Surveillance

There is considerable heterogeneity in the pattern of pertussis resurgence, not only related to heterogeneity in immunization and VCR practices, but also to the characteristics of epidemiological surveillance. Pertussis transmission is highest in the first two weeks after cough onset, a period when it is easily confused with other viral or bacterial respiratory diseases. This complicates both diagnosis and the timely application of preventive measures, such as chemoprophylaxis, when there is no or limited access to laboratory resources [1,2,3].

The WHO and CDC recommend that pertussis diagnosis should be confirmed through laboratory testing, including culture, polymerase chain reaction (PCR), and serology [40,41]. However, many MICs and LICs lack the resources necessary to implement these tests, contributing to the significant underreporting of the disease.

PCR has greatly improved the diagnosis of pertussis, especially in HICs and some MICs, where its introduction has led to more accurate epidemiological data [36,37,38,39,42,43,44]. Countries like Costa Rica, Argentina, and Brazil saw sharp increases in reported pertussis cases following the introduction of PCR-based diagnostic methods [37,38,39,42,43]. A similar situation was observed in other countries, like Russia and China. In Russia, in 2013 the PCR was used in 33 (38.8%) regions, increasing to 64 regions (75.3%) in 2017 [44]. In China, the introduction of nationwide guidance on PCR use for pertussis diagnosis in December 2023 may also be contributing to the observed increase in the identification of pertussis cases [29,30].

The improvement of the surveillance of community-acquired pneumonias (CAPs) was responsible for the better detection of pertussis in Egypt and South Africa. In Egypt, a study carried out between August 2014 and September 2017, with 400 children hospitalized for CAP, showed that *B. pertussis* was detected in 7.7% of infants under 4 months of age [45]. In South Africa, an increase in pertussis was also noted after the improvement of hospital surveillance of pneumonia and most cases occurred in children too young to receive the vaccine [46].

Since 1990s, serologic tests have been used in many HICs, especially to confirm pertussis diagnosis in adolescents and adults, even before the introduction of aP vaccines [47,48,49,50]. Berber et al. (2021) showed through a seroepidemiological study performed in European countries that pertussis was very common in adults (40–50 years) [51]. In MICs and LICs, serological tests are rarely used to confirm pertussis, but there is evidence that pertussis is common in adolescents and adults [52,53,54,55,56,57,58,59].

New diagnostic tests (multiplex PCR) capable of identifying multiple bacteria and viruses have been introduced in some countries, facilitating the rapid diagnosis of the causative agents of CAP. Through these tests, it is possible to verify that co-infections are common, particularly in children. In young infants, co-infection with RSV is the most common [60,61,62,63,64,65,66,67], and there is evidence that co-infection between *B. pertussis* and RSV may even worsen the clinical picture. Many pertussis cases may be ignored if physicians only search for RSV [60]. Other virus and bacteria can also cause co-infections in children and adults. In adults, co-infections with *B. pertussis* and influenza and other viruses also have been reported, but the detection of co-infections will depend on the seasonality, occurrence of outbreaks of pertussis, and capacity to detect viral and bacterial agents. Multiplex PCR tests are little used due to their high cost, but it is expected that in the future they may be more accessible for the early diagnosis of outbreaks and epidemics.

### 3.4. Characteristics of the Type of Vaccine Used in Primary Series

The analysis of these data clearly shows that pertussis resurgence was not related only to the type of vaccine used (wP or aP).

The latest WHO position paper on pertussis states that both wP and aP vaccines are effective in preventing the disease in the first year of life and that the vaccination of pregnant women should also be prioritized to protect infants too young to receive the first dose of the vaccine [2]. However, the assertion that “wP vaccines have greater efficacy and duration of protection compared to aP vaccines” should be revised.

The efficacy data for wP vaccines analyzed in the WHO position paper refer to vaccines that are no longer available and have been replaced in the last decades by wP vaccines never tested in efficacy trials. Additionally, even the old wP vaccines had variable efficacy, ranging from 36% to 96%, while the efficacy of aP vaccines has been consistently higher than 80% [2].

Several studies have been published in recent years showing that there are clear differences in the immune response to wP and aP vaccines. Although wP vaccines induce an immune response that appears closer in nature to that induced by natural infection and may have a greater impact on reducing respiratory tract colonization, according to data in non-human primate infection models, it is still unclear what the real role of this in the epidemiology of the disease. In an excellent review on the subject, Domenech de-Celles et al. (2024) [68] addressed the challenges related to the understanding of the immune response and the models used to assess the impact of different types of vaccines on the epidemiology and emergence of outbreaks. Many biases have been found in the studies that support the claims of greater protection and longer duration of protection offered by wP vaccines, such as different designs, use of different vaccines, lack of unvaccinated controls in efficacy studies, and heterogeneity of the populations and laboratory tests used to evaluate immune responses, among others. Immunity to pertussis is still little known and even natural infection does not protect for life. For these reasons, booster doses are necessary regardless of the vaccine used (wP or aP). More than preventing infection, it is crucial to reduce morbidity and mortality from pertussis. Acellular pertussis vaccines have proven their efficacy and effectiveness and can maintain protection until preschool age [1,2,7,69,70].

Although the wP vaccines currently in use can reduce the burden of the disease, there are few studies on their immunogenicity and effectiveness. The limited evidence on the immunogenicity of wP vaccines suggests that the duration of immunity is short, probably less than five years [71]. In one of the rare studies on the effectiveness of wP vaccines, conducted in Africa, the effectiveness was much lower than that offered by aP vaccines [72].

The concept that wP vaccines can induce long-duration protection also needs to be revised. The acellular pertussis vaccines for use in adolescents and adults were developed to protect these age groups that became more vulnerable to pertussis when HICs achieved high VCRs. These age groups obviously did not receive aP in their infancy. Serologic studies performed in HICs clearly showed that pertussis reinfections occur in adolescents and adults (47–51). In the last decade, several seroepidemiological surveys have also been carried out in MICs, such as Mexico, Russia, China, Iran, Cambodia, and Indonesia, which have revealed that asymptomatic pertussis infections occur 3 to 5 years after the last dose of wP vaccines, showing that the duration of protection offered by these vaccines also decreases over time [52,53,54,55,56,57,58]. Among these studies, the most interesting was carried out in Poland, because it was conducted in a population where half of the children had been vaccinated with a wP vaccine and the other half with an aP vaccine in the basic series. The reduction in anti-pertussis antibody titers occurred similarly in both groups, at five years after the preschool booster dose, indicating that the duration of protection provided by both vaccines was quite similar [73].

Data from Latin American countries demonstrated that adolescents and adults can be sources of pertussis infection to young infants [33,39,59]. Despite the limited access to serologic tests in this region, in Brazil, the largest use of PCR in some states showed that, in 2024, 38% of confirmed pertussis cases occurred in adolescents and 29% in adults. All 12 confirmed pertussis deaths registered in 2024 occurred in infants less than six months of age; from these, 75% were in infants of one or two months of age [35]. Besides the necessity to improve the VCR for children, health authorities have also emphasized the necessity to improve the Tdap VCR for pregnant women and health care professionals who have contact with infants.

## 4. Conclusions

The epidemiology of pertussis is extremely variable and is not related solely to the type of vaccine used (wP or aP). Incidence rates are influenced by multiple factors inherent to the bacteria (high transmissibility and silent circulation), vaccination schedules (number of doses and boosters), vaccination coverage, and access to laboratory resources to diagnose and implement control measures to prevent the rapid spread of the disease.

Periodic epidemics occur at intervals of 2 to 5 years, whenever there is an accumulation of susceptible individuals. The last pertussis epidemics occurred more than five years ago. During and immediately after the COVID-19 pandemic, a reduction in vaccination coverage was observed and millions of children did not receive the primary series and booster doses. In 2022, incidence rates increased in several countries. Apparently, countries that use aP vaccines have higher incidence rates of the disease, but it is not possible to compare the epidemiological data of countries with differing surveillance capacities and different criteria for the clinical and laboratory diagnosis of pertussis.

HICs offer better access to health services and laboratory resources that facilitate diagnosis in different age groups. In these countries, most pertussis cases are recorded in adolescents and adults and deaths are rare, occurring mainly in very young infants whose mothers did not receive Tdap during pregnancy. In MICs/LICs, although pertussis incidence rates are generally lower, only more severe cases are diagnosed and the number of deaths is much higher than that recorded in HICs.

Infant protection is a priority to reduce the risk of hospitalizations and deaths. It is essential to achieve a VCR > 90% in the first year of life and to vaccinate pregnant women with Tdap in each pregnancy. All children should be vaccinated without delay and booster doses are necessary, regardless of the vaccine used (wP or aP).

Children who were not vaccinated during the COVID-19 epidemic should receive booster doses to prevent outbreaks in daycare centers/schools.

Up-to-date and accurate epidemiological information is essential for taking immediate action. Therefore, it is essential to improve epidemiological surveillance by making highly sensitive tests available for diagnosing the disease, as well as educating doctors and laypeople about the risk of pertussis epidemics after the COVID-19 pandemic. Unfortunately, in the poorest regions, there is a lack of reliable data on the epidemiology of the disease and there is a delay in the investigation and dissemination of information. It is precisely in these regions that pertussis causes the most deaths.

## Figures and Tables

**Table 1:** Pertussis incidence rates by WHO region, 2014 to 2024 [10].

WHO Region	2023	2022	2021	2020	2019	2018	2017	2016	2015	2014
African	8.4	7.3	6.2	4.9	8.6	16.4	10.5	3.1	11.6	**17.6**
Eastern Mediterranean	5.5	2.3	**8.8**	1	3.2	5.4	3.3	3.3	2.6	3.9
European	**104.4**	6.8	3.4	23.7	74.7	66.1	68.1	81.1	67.1	72.2
American	6	3.2	6.6	20.1	11.5	23.5	29.4	28.6	32.9	**48.4**
South-east Asia	5.8	4.7	0.4	6.3	6	8.7	17.1	21.3	17.7	**28.5**
Western Pacific	26.4	21.7	5.8	6.1	**33.2**	27.9	14.6	16.2	18.5	10.3

Note: Incidence rates refer to the IR per 1,000,000 in total population. In bold the highest pertussis IR from 2014 to 2024.

**Table 2:** Pertussis incidence rates in selected HIC in the last 10 years, 2014–2023.

	Pertussis Incidence Rates Post-COVID-19 Pandemic					Pertussis Incidence Rates Pre-COVID-19 Pandemic				
Country	2023	2022	2021	2020	2019	2018	2017	2016	2015	2014
Australia	92.3	18.3	21.2	134.3	471.7	499.8	489.8	823.6	**939.9**	501.9
**Austria**	**305.6**	18.1	14.4	73.4	251.4	**248.5**	160.4	145.3	67	43.3
Belgium	89.3	6.9	1.4	10.7	95.6	118.5	134.9	**185.7**	106.7	133.9
Canada	36.5	5.4	0.8	25	67.3	45	**108.6**	107.2	97.6	42.9
**Croatia**	**1233.6**	0.5	1.8	2.5	14	**31.8**	19.6	19.1	13.6	-
Czechia	45.7	8.8	4.8	66	137	71.3	63.3	59.5	55.6	**239.7**
**Denmark**	**1018.6**	8.8	13.7	490.8	635.8	177.4	187.5	**364.9**	169.3	151.5
Netherlands	156.3	7.2	4.2	53.5	346.6	270.4	260.2	205.9	383.8	**526.1**
Spain	53.4	4.5	1.2	5.1	64.8	77.8	98.9	**104.7**	-	-
UK	13.7	1.2	0.8	18.2	68.7	51.3	76.8	**107.5**	79.6	62.4

Note: Incidence rates refer to the IR per 1,000,000 in total population. All countries adopted acellular pertussis vaccines before 2014. In bold, the highest pertussis IR reported before COVID-19 pandemic and in red, the highest IR reported in the last 10 years.

**Table 3:** Pertussis incidence rates in MICs and LICs, pre- and post-COVID-19 pandemic, in selected countries, 2014 to 2023 [10].

	Pertussis Incidence Rates Post-COVID-19 Pandemic					Pertussis Incidence Rates Pre-COVID-19 Pandemic				
Country	2023	2022	2021	2020	2019	2018	2017	2016	2015	2014
**Afghanistan (wP)**	**36.9**	8.2	2.7	-	-	**13.3**	0	0	12.8	0
Argentina (wP)	13.3	4.3	3.8	3.6	21.7	20.2	19.5	**38.4**	22.4	13
**Bolivia (wP)**	**67.5**	18.5	0	1	2.5	1.8	0.6	2.4	1	**6.9**
Chile (wP/aP) ^1^	21.6	0	1.5	3.2	18.5	36.1	45.7	41.2	40.7	**63**
**China (aP) ^2^**	**28.9**	26.9	6.7	3.1	21.2	**15.5**	7.4	4	4.8	2.5
Costa Rica (aP)	4.3	1	0.4	2	10.2	7.3	**11.8**	4.7	4.2	6.5
**Ecuador (wP)**	**10.3**	3.2	0	0.5	3.3	1.8	**3.6**	1	0.4	0.6
India (wP)	3.4	3.1	0.4	9	8.5	9.6	17.5	27.7	19	**35.6**
**Indonesia (wP)**	**7.7**	1.5	-	0.1	0.1	0.1	3.9	3.1	-	**8**
**Malaysia (aP)**	**34.2**	2.9	0.3	4	27.4	27.1	10.9	9.4	**30.1**	16.3
Mexico (aP)	1.4	0.5	0.2	2	6.9	6.3	6.7	8.4	**9.1**	8
**Papua New Guinea (wP)**	**177.1**	0.8	1	5.2	**48.1**	0	0	0	0	0
**Peru (wP)**	3.9	0.1	1.1	1.4	12.8	15.1	**19.5**	4.6	39	7.8
**Russian Fed. (wP/aP) ^3^**	**362.9**	21.9	7.6	41.5	**96.3**	71.2	37	56.5	44.4	32.5
**Serbia (wP)**	**209.8**	0.7	0.7	9	-	50	40.3	20.8	12.4	**38.9**
South Africa (aP)	4.2	**12.6**	9.1	1.7	9.3	**16.3**	1.8	0.7	1.8	4.1
Ukraine (wP)	18.7	0.8	2.1	23.3	51.5	49	54.6	**68.7**	53	-

Notes: Incidence rates refers to incidence 1 per 1,000,000 in total population. In bold, the year with the highest IR reported before the COVID-19-pandemic. In red, the highest IR reported in the last 10 years; ^1^ Chile introduced aP in routine schedule in 2018; ^2^ China introduced aP co-purified vaccines in routine schedule in 2015; ^3^ Russian Federation introduced aP in routine schedule 2022.

## Data Availability

All data were based in the references mentioned in the paper.

## Supplementary Materials

The following supporting information can be downloaded at: https://www.mdpi.com/article/10.3390/vaccines12121346/s1, Table S1: Vaccine coverage rates (%) for the third dose of pertussis vaccines in selected MICs/LICs, 2014–2023 [8].
