# Supplementary material for: Epidemiology of Pertussis After the COVID-19 Pandemic: Analysis of the Factors Involved in the Resurgence of the Disease in High-, Middle-, and Low-Income Countries
Source: Vaccines (Basel). 2024 Nov 28;12(12):1346. doi: 10.3390/vaccines12121346 (PMC11679829; doi:10.3390/vaccines12121346)
Supplement: Supplementary file 1 [file vaccines-12-01346-s001.zip › vaccines-3303058-supplementary.pdf]

Table S1: Vaccine coverage rates (%) for the third dose of pertussis vaccines in selected MICs/LICs, 2014–2023 [8].

Country	2023	2022	2021	2020	2019	2018	2017	2016	2015	2014
Afghanistan	60	58	55	61	65	**67**	63	65	64	63
Angola	54	42	45	51	57	63	56	59	59	58
Argentina	66	84	81	74	83	86	86	92	94	94
Brasil	90	77	68	77	70	87	83	89	96	93
Ecuador	70	70	72	70	**85**	**85**	**85**	83	78	83
Indonesia	83	91	67	77	85	85	84	83	87	**92**
Venezuela	54	43	56	54	64	60	66	84	**87**	78

Notes: In **bold**, the highest VCR in the last 10 years.
